# Supplementary material for: Malaria infection and anemia status in under-five children from Southern Tanzania where seasonal malaria chemoprevention is being implemented
Source: PLoS One. 2021 Dec 2;16(12):e0260785. doi: 10.1371/journal.pone.0260785 (PMC8638878; doi:10.1371/journal.pone.0260785)
Supplement: Attachment — Submitted filename: Reviewers comments.docx [file pone.0260785.s002.docx]

**Reviewers' comments**

My compliments to the authors, this is a well written manuscript about a properly designed and explained study. However, I have a few questions/remarks though:

**Title**
- The title was not fluent. So I wanted to suggest “Malaria infection and anemia status in underfive South Tanzanian children where seasonal malaria chemoprevention is being implemented” instead of “Malaria infection and anemia status in underfive children in an area initiating seasonal malaria chemoprevention in Southern Tanzania”.

-**Under introduction**

Justification and rationale for the study were not clearly stated. However, the introduction was more of information from literature. Please kindly review your introduction by elaborating on your justifications and rationales.

- Was the Ward randomization performed with a computer? Please elaborate.

- **Under study population**
- I will suggest you rephrase it (P6, line 127) to mean willingness of the parents or caregivers to consent since these infants and your children are ethnically ineligible to consent for the study themselves.

P6, line 128, please add the duration of stay in the study area.

- **Under malariometric study**
- Please cite (P7, lines 148-149).
- Please specify software program used and name of manufacturer (P7, line 155).
- Please specify the number of readings used in the calculation for average parasite density (P8, lines 170- 171).
- Please cite (P8, lines 174-175).
- **Under ethical consideration**
- Please cite (P8 line 194-195).
- **Under relationship between clinical presentation and malaria infection status**
- - The sentence is not clear (P12, lines 259-263).
- Please clearly state whether it is positively or negatively associated (P13, line 266).
- See Attachment for others minor errors
