# Supplementary material for: Malaria infection and anemia status in under-five children from Southern Tanzania where seasonal malaria chemoprevention is being implemented
Source: PLoS One. 2021 Dec 2;16(12):e0260785. doi: 10.1371/journal.pone.0260785 (PMC8638878; doi:10.1371/journal.pone.0260785)
Supplement: Attachment — Submitted filename: Malaria & Anemia Status-309082021.docx [file pone.0260785.s003.docx]

**Malaria infection and anemia status in underfive South Tanzanian children where seasonal malaria chemoprevention is being implemented**

**Authors**

Richard O. Mwaiswelo^1*^, Bruno P. Mmbando^2^, Frank Chacky^3^, Fabrizio Molteni^3^, Ally Mohamed^3^, Samwel Lazaro^3^, Sylvia F. Mkalla^4^, Bushukatale Samuel^5^, Billy Ngasala^5^_._

**Affiliation**

^1^Department of Microbiology, Immunology and Parasitology, Hubert Kairuki Memorial University, Dar es Salaam, Tanzania.

^2^Departments of Statistics, National Institute for Medical Research, Tanga Centre, Tanga, Tanzania.

^3^National Malaria Control Programme, Ministry of Health, Community Development, Gender, Elderly and Children, Dodoma, Tanzania.

^4^Directorate of Research, Coordination and Promotion, Tanzania Commission for Science and Technology, Dar es Salaam, Tanzania.

^5^Department of Medical Parasitology and Entomology, Muhimbili University of Health and Allied Sciences, Dar es Salaam, Tanzania

*Corresponding author: [richiemwai@yahoo.com](mailto:richiemwai@yahoo.com) (ROM)

# Abstract

**Background:** Malaria and anemia remain major public health challenges in Tanzania. Household socioeconomic factors are known to influence these conditions. However, it is not clear how these factors influence malaria transmission and anemia in Masasi and Nanyumbu Districts. This study presents findings on malaria and anemia situation in underfive children and its influencing socioeconomic factors in Masasi and Nanyumbu Districts, surveyed as part of an ongoing seasonal malaria chemoprevention operational study.

**Method**s: A community-based cross-sectional survey was conducted between August and September 2020. Finger-prick blood samples collected from children aged 3-59 months were used to test for malaria infection using malaria rapid diagnostic test (mRDT), thick smears for determination of asexual and sexual parasitemia, and thin smear for parasite speciation . Hemoglobin concentration was measured using HemoCue spectrophotometer. A structured questionnaire was used to collect household socioeconomic information from parents/caregivers of screened children. Prevalence of malaria was the primary outcome. *Chi*-square tests, *t*-tests, and logistic regression models were used appropriately.

**Results:** Overall mRDT-based malaria prevalence was 15.9% (373/2340), and was significantly higher in Nanyumbu (23.7% (167/705) than Masasi District (12.6% (206/1635), *p*<0.001. Location (Nanyumbu), no formal education, household number of people, household number of underfives, not having a bed net, thatched roof, open/partially open eave, sand/soil floor, and low socioeconomic status were major risks for malaria infection. Some 53.9% (1196/2218) children had anemia, and majority were in Nanyumbu (63.5% (458/705), *p*<0.001. Location (Nanyumbu), mRDT positive, not owning a bed net, not sleeping under bed net, open/partially open eave, thatched window, sex of child, and age of child were major risk factors for anemia.

**Conclusion:** Prevalence of malaria and anemia was high and was strongly associated with household socioeconomic factors. Improving household socioeconomic status is expected to reduce the prevalence of the conditions in the area.

**Key words:** Malaria, Anemia, underfive children, socioeconomic factors, Tanzania.

Introduction
Malaria is an infection of major public health importance in sub-Saharan Africa (SSA) [1,2]. It is one of the leading causes of morbidity and mortality in SSA [3]. Children aged below five years is among the groups most affected by the infection [1,3,4]. Anemia is one of the major complications of malaria infection [5]. Malaria infection causes hemolysis of parasitized and non-parasitized erythrocytes, and bone marrow dyserythropoiesis that compromises rapid recovery from anemia [5–7]. Consequently, in high malaria endemic settings majority of infants and young children, and many older children and adults have anemia of varying degrees [7]. In these settings severe life-threatening malarial anemia requiring blood transfusion in young children is among the major cause of hospital admission, especially during the rainy season [7–9]. Anemia also contribute directly or indirectly to a significant proportion of malaria-related deaths [7,8,10,11]. Of note, effective use of malaria interventions prevent malaria morbidity including anemia, and malaria-related mortality [7].

Malaria incidences, transmission rate, and vector distribution vary markedly across and within countries, or even over short distances between neighboring villages and even within a single settlement as a result of small variation in risk factors [12–14]. Understanding these risk factors may streamline the implementation and evaluation of the impact of malaria interventions. Malaria transmission is highly dependent on temperature, humidity, and rainfall [14,15]. Besides climatic factors, the transmission is also influenced by other factors including socioeconomic factors, and the availability and accessibility of malaria control tools [14,16]. Socioeconomic factors such as level of education of the head of household, occupation of the head of household, household income, type and quality of house, and residential setting it being rural or urban have been found to determine malaria transmission in endemic settings [13,16]. For instance, low level of education of the household head, low household income, poor quality of the house, late outdoor activities, and rural settings have been associated with high burden of malaria infection [13,16–19]. The burden of malaria is also influenced by the proximity of human settlements to vector breeding sites, and presence of domestic animals near homesteads [14,17,18]. On the other hand, availability and accessibility of the control tools significantly influence the transmission of malaria. Major tools for control of malaria in Tanzania include vector control using insecticide treated bed nets (ITNs), indoor residual spraying, chemoprophylaxis in pregnant women, and management of clinical cases using artemisinin-based combination therapy (ACT) [20,21]. Of the available vector control tools, ITNs is the most used tool [20–23]. The ITNs use is estimated to reduce mortality rates by 55% in children aged below five years in sub-Saharan Africa, and incidences of malaria cases by 50% in various settings [20,24]. To have maximum protection at the household and the entire community, high coverage with ITNs coupled with its proper use is a prerequisite [25], and one bed net per two persons per household is recommended [25,26]. Importantly, prior to the introduction of additional malaria control tools i.e. seasonal malaria chemoprevention (SCM), understanding the ITNs coverage in an area is crucial so as to allow for effective evaluation of the impact of the additional tools. This article therefore, presents findings to enlighten the baseline malaria and anemia situation and the influencing socioeconomic factors in Masasi and Nanyumbu districts, as part of an ongoing SCM study.

# Materials and Methods

Study area
The study was carried out in Nanyumbu and Masasi Districts, Mtwara Region. Nanyumbu has a total area of 5,204 square km, and is divided into 14 wards and 89 villages, whereas Masasi has a total area of 3,829.9 square km and is divided into 22 wards and 159 villages. Nanyumbu District has a total population of 166,277, and Masasi has 269,590. Of the population, 115,735 and 152,609 in Nanyumbu and Masasi Districts, respectively, live in rural areas. The districts had a projected population of 82,740 children aged 3-59 months in 2019, 31,564 in Nanyumbu and 51,176 in Masasi District. By 2018, there were 44,319 and 73,998 households in Nanyumbu and Masasi District, respectively. Nanyumbu has 19 health facilities and Masasi has 40. The major economic activities in Masasi and Nanyumbu Districts include subsistence farming, trade, fishing, and small-scale mining.

Nanyumbu and Masasi Districts have annual rainfall averaging 939 mm and temperature of 25.4°C. The rainy season in Nanyumbu and Masasi Districts is between January and April, and accounts for > 60% of the average annual rainfall. Both districts have high seasonal malaria transmissions where > 60% of cases occur between March and July. *P. falciparum* is the predominant malaria parasite, and *Anopheles arabiensis* the major vector. Insecticide treated bed-nets and diagnosis and treatment with antimalarial drugs are the major malaria control measures in the area.

Study design
This was a community-based cross-sectional malariometric and socioeconomic survey with wards used as sampling units. Health facilities nearest to the centroid of the selected wards were identified (satellite facilities) and the catchment population estimated. A study village(s) within the health facility catchment area was identified (satellite villages) and demographic survey was carried out to determine the population structure, and the number of possible study participants as well as socio-economic characteristics of the households. Malariometric survey was conducted in all the selected villages to establish baseline malariometric indices including type of malaria parasite, prevalence of malaria infection, haemoglobin levels, and assessment of nutritional status.

Study population
Both febrile and afebrile children of both sexes were involved in the malariometric survey, whereas heads of the households were involved in the household survey. The study inclusion criteria for malariometric survey were being a child aged 3-59 months, living within the catchment area, and willingness to participate in the study. The exclusion criteria were presence of severe illness, and history of intake of antimalarial drug within the previous 30 days or being under cotrimoxazole prophylaxis.
Inclusion criteria for the household survey included living within the catchment area, willingness to participate in the study, and having a child who has participated in the malariometric survey. The head of the household was defined as a person who is perceived by household members to be the primary decision maker in the family, and the household was defined as individuals living together and taking meals from a common cooking facility [27]. In the absence of the head of the household, a responsible person above 18 years who had been appointed by the family was interviewed.

Procedures for data collection
Research assistants including clinicians, laboratory technicians, and community health workers were trained on the study objectives and procedures prior to data collection. Social mobilization of the participating communities was organized through local meetings and announcements in the houses of worships prior to the start of the study to explain the purpose of the study, its benefit to the community and the importance of community member’s participation.

**Malariometric survey:** The survey was carried out at the satellite health facilities whereby all the febrile and afebrile children of the required age were brought for clinical and laboratory assessments. The survey was conducted for three days at each of the selected ward.

Clinical assessment involved taking history of clinical symptoms, history of antimalarial drugs consumption within past 30 days, use of cotrimoxazole as chemoprophylaxis, clinical examination including measurement of axillary temperature to determine presence of fever, and anthropometric measurements. The anthropometric measurements including height/length and weight were recorded for each child by the same investigator using standard techniques. The height of the children was recorded to the nearest 0.1 cm using anthropometric height rod. For children below 24 months of age, length was measured using infant-meter. The weight measurements were recorded to the nearest 100 g using SECA electronic weighing scale. Repeated measurements were made for 20 children to periodically correct for the intra observer error. The z-scores for three indices, i.e., weight-for-age, height-for-age, and weight- for-height were then calculated using computer software. The median values of the National Bureau Statistics reference data were used as standards. Additionally, the mid-arm circumference was measured for every child assessed.

Laboratory assessment involved collection of finger-prick blood samples that were used to assess presence of malaria infection using malaria rapid diagnostic test (mRDT), prepare thick film for microscopy to assess density of asexual parasitemia and gametocytemia, and thin film for asexual parasite species determination. The blood samples were also used to measure haemoglobin concentration.
Thin films were fixed using absolute methanol. Both thin and thick blood films were air-dried, stained using 3% giemsa for 1 hour, and examined for malaria parasites at 100 high power fields under immersion oil. Parasite density was determined by counting the number of parasites present per 200 white blood cells (WBC) on a thick smear, and the obtained number was multiplied by 40 assuming a WBC count of 8,000 per milliliter of blood. A blood slide was considered negative if no parasite was seen after examining 100 fields. Slides were read independently by 2 laboratory technicians, who were unaware of the ward the slides came from and the mRDT results. In case of a discrepancy (positive versus negative or a difference in parasite density greater than 30%), a third reading was requested and the average parasite density of two or three readings was used. Microscopists at the National Institute for Medical Research (NIMR), Tanzania read 10% of the slides for quality control.
Hemoglobin concentration was measured using a portable spectrophotometer, HemoCue Hb 301+ (HemoCue AB, Ängelholm Sweden), with a precision of +/- 0.3 g/dL. The HemoCue was calibrated every morning using a control cuvette at 16.0+/-0.3 g/dL according to manufacturer’s instruction. Anaemia was classified as haemoglobin level < 11g/dL (mild), < 7g/dL (moderate) and < 5g/dL (severe).

**Socioeconomic survey:** The survey was carried out in catchment villages of the selected wards. The Community Health Workers (CHWs) conducted the household survey using a structured questionnaire with both close and open ended questions. The CHWs administered the questionnaire. The questionnaire inquired information on demographic characteristics of the household, socioeconomic status including house type and household assets, awareness on malaria infection and its control measures, and ownership, usage, and perception of the household on the available major malaria control tools in the districts particularly insecticide treated bed nets (ITN). The use of ITN was assessed through asking if a child slept under ITN the previous night, and the CHWs confirmed the presence of the bed net.

Study outcomes
The primary outcome was the prevalence of malaria infection defined as the presence of *P. falciparum* asexual parasitaemia at any density. Secondary outcomes include: i) mild, moderate, or severe anaemia defined as an haemoglobin concentration of 11 g/dL, 8 g/dL, and 5 g/dL, respectively; ii) clinical presentations that predict malaria infection (iii) socioeconomic factors influencing malaria transmission (iv) socioeconomic factors influencing anemia.

Ethical consideration
The study was conducted in accordance with the declaration of Helsinki, good clinical practices, and regulations in Tanzania. The study was approved by the ethics committees of the Muhimbili University of Health and Allied Sciences, Tanzania.

Members of the study team held meetings with community, administrative, and religious leaders to explain the aims and activities of the study and sought community approval. Project CHWs then visited the households to explain the aim of the study, provided information sheets, and sought signed consent from parents or guardians of the children who were involved in the malariometric survey. The parents/guardians also provided the consent to participate in the socioeconomic survey.

## Statistical analysis

This was a baseline survey for seasonal malaria chemoprevention (SMC) study, thus sample size calculation was based on the estimations made for SMC, and is presented elsewhere. Briefly, there is variation in malaria incidence between the two districts, with three years (2016-2018) average of 309 and 222.5 cases per 1000 in Nanyumbu and Masasi District, respectively. Malaria control in both districts relies mainly on long lasting insecticides nets and prompt diagnosis and treatment with ACTs for uncomplicated *falciparum* malaria. Thus a total of 20 wards were selected into the study, and in each ward 106 children were to be assessed with a power of 80% and an alpha (type one error) of 0.05. An attrition rate of 20% was considered resulting into a sample of 128 children per evaluable ward. Similar to the number of children expected to participate in malariometric survey, 128 households had to be assessed for socioeconomic survey assuming each household contributed one child.

Data were collected electronically in open data kit (ODK) software using tablets computers. Spatial data (point coordinates for households and boundaries of villages and other features from the study areas) were collected during the household’s visits using Global Positioning System (GPS) inbuilt in mobile phones and was downloaded into Quantum Geographical Information System (QGIS), a free software for spatial analysis and mapping. Quality control and assurance of the data were maintained at all stages of data collection to archiving. Back-ups of data was made on a daily basis onto external hard disks and stored in a secured place separately from the building hosting data management section. Source documents (in case of paper based) were achieved and sprayed regularly to keep them away from destruction by insects for the duration specified in the protocol. Data were cleaned and analysed using STATA version 13 statistical package. Categorical variables were presented in proportions and compared using *χ*2 tests while continuous variables were summarised as means with standard deviations and compared using t-test. Mean values and 95% confidence intervals (95%CI) for different variables were compared between mRDT positive and negative individuals using t-test. A χ2 tests was also used to assess the trend of continuous and categorical variables by age groups. Logistic regression analysis was used to assess the association of mRDTs positivity with different parameters. A *p*-value < 0.05 was considered statistically significant.

# Results

## Baseline characteristics and distribution of malaria infection by age and district

A total of 2340 children, 1635 (69.9%) from Masasi and 705 (30.1%) Nanyumbu Districts were screened for malaria parasites using both malaria rapid diagnostic test (mRDT) and microscopy. The baseline characteristics of the screened children are presented in Table 1. Of the screened children, 373/2340 (15.9%) were malaria positive by mRDT and 212/2330 (9.1%) were positive by microscopy. The prevalence of malaria was significantly higher in Nanyumbu (23.7% (167/705 than in Masasi district (12.6% (206/1635), (*p*<0.001).

**Table 1:** **Baseline characteristics of the participants**

| **Variable** | **Overall** | **Masasi** | **Nanyumbu** | **Test** |
| --- | --- | --- | --- | --- |
| Age, median (IQR), years | 2.25 (1.12-3.43) | 2.23 (1.18-3.45) | 2.24 (1.27-3.40) | *p*=0.855* |
| Sex, female, n (%) | 1215 (51.92) | 865 (52.91) | 350 (49.65) | *p*=0.148 |
| Weight (kg), mean (SD) | 11.45 (2.99) | 11.52 (3.02) | 11.29 (2.91) | *p*=0.086^δ^ |
| Height (cm), mean (SD) | 81.72 (13.18) | 81.99 (12.96) | 81.13 (13.64) | *p*=0.144^δ^ |
| mRDT positive, n (%) | 373 (15.94) | 206 (12.60) | 167 (23.69) | *p*<0.001 |
| Blood slide positive, n (%) | 212 (9.10) | 106 (6.52) | 106 (15.08) | *p*<0.001 |
| Mean parasite density/μL, (95%CI) | 193 (137- 272) | 458 (268-785) | 81 (57-117) | *p*<0.001 |
| Mean Hb concentration, g/dL, (SD) | 10.94 (1.42) | 11.1 (1.43) | 10.6 (1.34) | *p*<0.001^δ^ |

*Kruskal-Wallis equality of population rank test; ^δ^two sample t-test.
Distribution of malaria infection by age groups and district is presented in Table 2. The prevalence of malaria was highest in the age group of 48-59 months both in the overall study population and by districts. Trend analysis showed that the prevalence of malaria was statistically significantly increasing with age in the overall study population and by district.

Table 2: Distribution of malaria infection by age group and district

| **Age group (months)** | **mRDT malaria positivity** | | | **Test, *p*-value** |
| --- | --- | --- | --- | --- |
|  | **Overall (%)** | **Masasi (%)** | **Nanyumbu (%)** |  |
| 3-11 | 49/474 (10.3) | 26/337 (7.7) | 23/137 (16.8) | *x*^2^=8.65, *p* = 0.003 |
| 12-23 | 63/511 (12.33) | 28/333 (8.41) | 35/178 (19.66) | *x*^2^=13.59, *p* <0.001 |
| 24-35 | 89/482 (18.46) | 52/336 (15.48) | 37/146 (25.34) | *x*^2^=6.58, *p* = 0.010 |
| 36-47 | 86/425 (20.24) | 48/294 (16.33) | 38/131 (29.01) | *x*^2^=9.03, *p* = 0.003 |
| 48-59 | 82/352 (23.30) | 48/241 (19.92) | 34/111 (30.63) | *x*^2^=4.88, *p* = 0.027 |
| **Trend test** | *x*^2^=27.1, *p*<0.001 | *x*^2^=16.3, *p*<0.001 | *x*^2^=11.2, *p*<0.001 |  |

## Relationship between clinical presentation and malaria infection status

The relationship between clinical presentation and mRDT positivity was assessed in both symptomatic and asymptomatic children. The likelihood of having positive mRDT results was statistically significantly higher in children with symptoms than in those without symptoms, Table 3.

Table 3: Relationship between clinical presentation and mRDT positivity

| **Clinical presentation** | | **mRDT positive (%)** | **Test, *p*-value** |
| --- | --- | --- | --- |
| Fever | Yes | 148/214 (69.2) | *x*^2^=482, *p*<0.001 |
|  | No | 222/2048 (10.8) |  |
| History of fever in past 24 hours | Yes | 245/284 (86.3) | *x*^2^=1200, *p*<0.001 |
|  | No | 125/1934 (6.5) |  |
| Use of antimalarial drugs within past 14 days | Yes | 201/478 (42.0) | *x*^2^=282, *p*<0.001 |
|  | No | 169/1740 (9.7) |  |
| Headache | Yes | 101/107 (94.4) | *x*^2^=488, *p*<0.001 |
|  | No | 269/2111 (12.7) |  |
| Cough | Yes | 119/355 (33.5) | *x*^2^=86.2, *p*<0.001 |
|  | No | 251/1863 (13.5) |  |
| Diarrhea | Yes | 23/41 (56.1) | *x*^2^=47.0, *p*<0.001 |
|  | No | 347/2177 (15.9) |  |
| Vomiting | Yes | 58/64 (90.6) | *x*^2^=259, *p*<0.001 |
|  | No | 312/2154 (14.5) |  |
| Abdominal pain | Yes | 88/92 (95.7) | *x*^2^=430, *p*<0.001 |
|  | No | 282/2126 (13.3) |  |

## Relationship between socioeconomic factors and malaria infection

Socioeconomic factors and its relationship with malaria infection are presented in Table 4. Univariate regression model analysis indicated that district (Nanyumbu), level of education (primary and secondary education) of the head of household, having five or more occupants in the household, having more than one underfive children in the household, ownership of bed nets, only parents using bed nets, iron sheet roof, floor type, and household level of economic status were the factors statistically significantly associated with malaria infection. Multivariate model analysis indicated that district (Nanyumbu), primary and secondary education, number of people more than 5, more than one underfive children, ownership of bed net, and medium and upper economic status were the only factors associated with malaria infection.

Table 4: Relationship between household socioeconomic factors and malaria infection

| **Variable** | |  | Univariate | Multivariate (n=1203) |
| --- | --- | --- | --- | --- |
|  |  | **Prevalence (%)** | **OR (95%CI); *p*-value** | **aOR (95%CI) *p*-value** |
| District | Masasi | 206/1652 (12.5) | 1 | 1 |
|  | Nanyumbu | 168/716 (23.5) | 2.15 (1.71-2.70); <0.001 | 1.9 (1.34-2.68); <.001 |
| Sex of head | Male | 197/1,237 (15.9) | 1 |  |
|  | Female | 66/429 (15.4) | 0.96 (0.71-1.30); 0.791 |  |
| Marital status | Single | 72/438 (16.4) | 1 |  |
|  | Married | 176/1,137(15.5) | 0.93 (0.69-1.26); 0.640 |  |
|  | Separates | 15/90 (16.7) | 1.02 (0.55-1.87); 0.958 |  |
| Education level | None | 36/142(25.3) | 1 | 1 |
|  | Any primary | 217/1,374(15.8) | 0.55 (0.37-0.83); 0.004 | 0.53 (0.31-0.90); 0.018 |
|  | Any Secondary | 10/150 (6.7) | 0.21 (0.10-0.46); <.001 | 0.33 (0.13-0.81); 0.016 |
| Occupation | Farming | 245/1,548(15.8) |  |  |
|  | Petty trader | 18/105 (17.1) | 1.10 (0.65-1.86); 0.721 |  |
| No. of people | 2-5 people | 197/1,322 (14.9) | 1 | 1 |
|  | >5 people | 66/339 (19.5) | 1.38 (1.01-1.88); 0.040 | 1.52 (1.02-2.25); 0.039 |
| No. children <5 years | One | 190/1,322 (14.3) | 1 | 1 |
|  | More than One | 70/284 (24.6) | 1.96 (1.43-2.68); <.001 | 1.39 (0.92-2.10); 0.120 |
| Ownership of bed nets | Yes | 185/1,412 (13.1) | 1 | 1 |
|  | No | 78/254(30.7) | 2.95 (2.16-4.03); <.001 | 2.53 (1.60-4.0); <.001 |
| Who use bed nets | Child plus others | 179/1,394 (12.8) | 1 |  |
|  | Parents only | 6/18 (33.3) | 3.39 (1.25-9.18); 0.010 |  |
| Roof type | Thatch | 140/595 (23.5) | 1 |  |
|  | Iron sheet/tiles | 123/1,071 (11.5) | 0.42 (0.32-0.52); <.001 |  |
| Eave type | Open | 184/1,226 (15.0) | 1 |  |
|  | Partially open | 30/86 (34.9) | 0.30 (0.17-0.52); <.001 |  |
|  | Closed | 49/354 (13.8) | 0.32 (0.20-0.53); <.001 |  |
| Window type | No window | 72/468(15.4) | 1 |  |
|  | Wire mesh | 60/388(15.5) | 1.01 (0.70-1.46); 0.974 |  |
|  | Wood/iron sheet | 96/638(15.1) | 0.97 (0.70-1.36); 0.877 |  |
|  | Thatch | 35/172(20.3) | 1.41 (0.90-2.20); 0.136 |  |
| Floor type | Soil/sand | 192/1,069 (18.0) | 1 |  |
|  | Cement | 71/597 (11.9) | 0.62 (0.46-0.83); 0.001 |  |
| Presence of electricity | Yes | 14/127 (10.2) |  |  |
|  | No | 249/1,539 (16.2) | 1.56(0.88-2.76); 0.126 |  |
| Social economic status | Low | 83/409 (20.3) | 1 | 1 |
|  | Medium | 50/409 (12.2) | 0.55 (0.37-0.80); 0.002 | 0.54 (0.36-0.83); 0.005 |
|  | Upper | 50/421 (11.9) | 0.53 (0.36-0.78); 0.001 | 0.41 (0.25-0.66); <.001 |

OR=odds ratio; aOR=adjusted odds ratio; 95%CI=95% confidence interval

## Prevalence of anemia and its distribution in relation to malaria infection status

A total of 53.9% (1196/2218) of screened children had anemia, of which 49.4% (748/1513) were in Masasi and 63.5% (458/705) in Nanyumbu District, (*x*^2^=38.5, *p*<0.001). Of the children with anemia, 95.9% and 4.1%, had mild and moderate anemia, respectively. The prevalence of malaria by age groups in the two districts (Panel A) and that of anemia by age groups in relation to malaria infection status in Masasi (Panel B) and Nanyumbu District (Panel C) are presented in Figure 1. The prevalence of anemia was significantly higher in children with mRDT positive results than in those with negative results both in both in Masasi (61.1% (124/203 *vs* 48.0% (636/1326) *x*^2^=12.1, *p*<0.001) and Nanyumbu District (76.8% (129/168) *vs* 59.9% (328/548), *x*^2^=16.0, *p*<0.001). The prevalence of anemia was significantly decreasing with an increase in age.

Figure 1: Prevalence of mRDT positives (A) and anaemia (B) by district and by age group. The open bars is Masasi, filled bars in Nanyumbu District while line segments presents 95%CIs.

## Relationship between socioeconomic factors and anemia

The relationship between household socioeconomic factors and anemia in the study population is presented in Table 5. Using the univariate regression model analysis, the participant’s district, mRDT positivity, ownership of bed nets, eave type, sex of the child, age of the child and thatched window were the factors associated with anemia in children. Multivariate regression analysis indicated that only district, mRDT positivity, sex of child, age of child and floor type were the factors associated with anemia.

Table 5: Household socioeconomic factors associated with anemia

| **Variable** | | **Univariate** | | **Multivariate** |
| --- | --- | --- | --- | --- |
|  |  | **Prevalence (%)** | **OR (95%CI); *p*-value** | **aOR (95%CI); *p*-value** |
| District | Masasi | 748/1513 (49.4) | 1 | 1 |
|  | Nanyumbu | 448/705 (63.5) | 1.78 (1.48-2.15); <0.001 | 1.97 (1.55-2.50); <0.001 |
| mRDT results | Negative | 944/1848 (51.1) | 1 | 1 |
|  | Positive | 252/370 (68.1) | 2.05 (1.61-2.60); <0.001 | 2.34 (1.72-3.19); <0.001 |
| Marital status | Single | 240/421 (57.0) | 1 |  |
|  | Married | 580/1056 (54.9) | 0.92 (0.73-1.15); 0.467 |  |
|  | Separated | 42/89 (47.2) | 0.67 (0.43-1.07); 0.09 |  |
| No. of people | 2-5 | 684/1251 (54.7) | 1 |  |
|  | >5 | 175/312 (56.1) | 1.06 (0.82-1.36); 0.654 |  |
| Ownership of bed net | Yes | 715/1327 (53.9) | 1 |  |
|  | No | 147/240 (61.2) | 1.35 (1.02-1.79; 0.035 |  |
| Who use bed net | None | 147/240 (61.2) | 1 |  |
|  | All | 705/1310 (53.8) | 0.74 (0.56-0.98); 0.033 |  |
|  | Parents only | 10/17 (58.8) | 0.904 (0.33-2.46); 0.843 |  |
| Roof type | Thatch | 329/575 (57.2) | 1 |  |
|  | Iron sheet | 533/992 (53.7) | 0.87 (0.71-1.07); 0.181 |  |
| Eave type | Open | 61/85 (71.8) | 1 |  |
|  | Partially open | 174/302 (57.6) | 0.53 (0.32-0.91); 0.018 |  |
|  | Closed | 627/1180 (53.1) | 0.45 (0.27-0.73); <0.001 |  |
| Window type | No window | 243/424 (57.3) | 1 |  |
|  | Wire mesh/gauze | 192/364 (52.7) | 0.83 (0.63-1.10); 0.199 |  |
|  | Wood/iron sheet | 345/608 (56.7) | 0.98 (0.76-1.26); 0.856 |  |
|  | Thatch | 82/171 (47.9) | 0.69 (0.48-0.98); 0.038 |  |
| Floor type | Soil/sand | 574/1012 (56.7) | 1 | 1 |
|  | Cement | 288/555 (51.9) | 0.82 (0.67-1.01); 0.066 | 0.77 (0.61-0.98);0.033 |
| Sex of child | Male | 629/1072 (58.7) | 1 | 1 |
|  | Female | 567/1146 (49.5) | 0.69 (0.58-0.82); <0.001 | 0.63 (0.51-0.79); 0.001 |
| Age of child in years | 3-11 | 341/450 (75.8) | 1 | 1 |
|  | 12-23 | 333/484 (68.8) | 0.70 (0.53-0.94); 0.017 | 0.64 (0.45-0.91); 0.014 |
|  | 24-35 | 200/447 (44.7) | 0.26 (0.19-0.35); <0.001 | 0.21 (0.15-0.30); <0.001 |
|  | 36-47 | 158/406 (38.9) | 0.20 (0.15-0.28); <0.001 | 0.18 (0.13-0.27); <0.001 |
|  | 48-59 | 118/335 (35.2) | 0.17 (0.12-0.24); <0.001 | 0.13 (0.09-0.19); <0.001 |
| Economic status | Low | 223/395 (56.5) | 1 |  |
|  | Medium | 214/383 (55.9) | 0.98 (0.74-1.30); 0.870 |  |
|  | Upper | 214/386 (55.4) | 0.96 (0.72-1.127); 0.775 |  |

OR=odds ratio; aOR=adjusted odds ratio; CI=confidence interval

# Discussion

Malaria is still an infection of major public health importance in Tanzania, especially among underfive children [20,22]. This study provides an important information on the prevalence of malaria infection and anemia among underfives and the associated household socioeconomic factors in Masasi and Nanyumbu Districts, the area where malaria transmission is highly seasonal, and a seasonal malaria chemoprevention operational research is ongoing. Despite the collection of data during the dry season, the overall prevalence of malaria in this area was high at 15.94%, and it was two folds higher than the national average of 7.3% [20]. But malaria prevalence in this study area was lower than that in Kakonko (30.8%) and Nanyamba (19.1%) Districts, Tanzania [20]. Of the socioeconomic factors assessed in relation to malaria infection, location particularly living in Nanyumbu District, having no education, household having more than five people, household having more than one underfive children, parents only sleeping under the bed net, not owning a bed net, thatched roof, sand/soil floor, open/partial open eave, and low socioeconomic status of the household were significantly associated with an increased risk of malaria infection. Similar factors have been associated with an increased risk of malaria infection in other parts of Tanzania [13,28–31], and in other countries including Botswana [18], Cameroon [32], Ethiopia [33,34], Equatorial Guinea [35], India [36], Kenya [37], and Rwanda [38,39]. The level of education affects the overall knowledge and ability of an individual to make decision, and also has effect on occupation and income. In this study, probably having no education led to poor decision making towards malaria control measures, and or poor income thus could not afford the control tools such as bed net, hence increasing the risk of infection. Large number of people in the house/room leads to a higher production of carbondioxide and other volatile gases which are attractive to mosquitoes thus increasing the number of mosquitoes in the house, and also many people are exposed to infectious bites per night [40–42]. Open/partial open eaves allows for easy entry of mosquitoes into the house, whereas thatched roof and sand/mud floor provides conducive microhabitat for mosquito resting [42], thus increasing the risks of infectious mosquito bite to the household occupants.
Nonetheless, in this study occupation, window type and electricity were not associated with malaria infection. Contrarily, in other studies [36,37,42], these factors were associated with malaria infection. Occupation and electricity failed to have significant influence on malaria infection probably because nearly all of the household heads had the same occupation, peasants, and likewise most of the households (92.4%) had no electricity. On the other hand, malaria prevalence in Nanyumbu District was nearly two folds higher than that of Masasi District. It is however not clear why Nanyumbu had much higher malaria prevalence than Masasi. All the assessed socioeconomic factors were not statistically significantly different between the two districts. However, the differences in malaria prevalence may probably be attributed to differences in transmission dynamics between the two districts including mosquito abundance, the factor which was not assessed in this study.

The prevalence of malaria was statistically significantly increasing with an increase in age. Similar findings have been reported in other studies [16,35,38,43,44]. The low infection rate among neonates and young infants might be attributed to the passive immunity infants acquire from their mothers [45,46], and the fetal hemoglobin (hemoglobin F) [45]. The passive immunity protects the infants against many infections including malaria, but as they grow this immunity tends to wane off and thus children become prone to the infection [45,46]. Hemoglobin F is known to poorly support *Plasmodium* parasite survival [45]. Furthermore, probably at the younger age many infants and younger children sleep under the bed nets, and normally under the protection of their parents who can control their sleeping behavior. But as they grow they stop sleeping with their parents, and therefore, even though they might be sleeping under the bed net, it becomes difficult to control their sleeping behavior, and thus expose themselves to the mosquito bite.

In this community-based study clinical presentations were strongly associated with mRDT positivity. Of the clinical presentations, abdominal pain (95.7%), headache (94.4%), vomiting (90.8%), history of fever in the past 24 hours (86.3) and fever (69.3%) were the strongest predictors of mRDT positivity. Other studies have also reported fever [47,48], history of fever [48], headache [48], and cough [35] to be the predictors of malaria infection. Interestingly, being a community based study it was expected that there would be no strong association between clinical presentations and mRDT positivity. However, in malaria endemic settings, asymptomatic malaria infection is common particularly in older children and adults who normally have developed partial immune against the infection, the immunity which normally develops after years of exposure to the infection. But since the children in this study were under the age of five years, probably they had not developed enough immunity to partially protect and lender them asymptomatic upon the infectious bite.

Anemia is one of the major complications of malaria infection, contributing directly or indirectly to hospitalization and deaths in young children [8–10]. More than half of the screened children in this study had anemia, and majority of them (95.9%) had mild anemia. Socioeconomic factors including being a resident of Nanyumbu District, mRDT positive results, ownership of bed nets, not sleeping under bed net, open/partially open eave, no windows, thatched window, sand/soil floor, male sex, and age of child were significantly associated with anemia. The same factors have shown to be associated with anemia in other studies [16,37,43,44]. Interestingly, whereas in other studies having no windows was not associated with malaria infection, in this study it was associated with increased risk of infection. Contrary to the findings in other studies [32,39], in this study socioeconomic status and number of people in the household were not associated with anemia. Conversely, the prevalence of anemia was much higher in Nanyumbu (63.9%) than in Masasi District (49.6%). Malaria infection may probably be the reason for higher prevalence of anemia in Nanyumbu as the infection was more prevalent in Nanyumbu District than Masasi District.
The prevalence of anemia was significantly higher in children with mRDT positive results than in those with negative results. Studies in Ghana [43,44], Kenya [16] and Rwanda [38,39] have presented similar findings. On the other hand, whereas the prevalence of malaria was increasing with an increase in age, the prevalence of anemia was decreasing with an increase in age. Similar findings have been reported in other studies [44,49]. The decrease of anemia with an increase in age probably indicated that at younger age, neonates and young infants are not breastfed adequately, and thus nutrients obtained in the milk are not adequate. But as these infants grow and start to feed on solid food they get more nutrients that improves their hemoglobin level, hence reduce prevalence of anemia. Interestingly, more than half of the mRDT negative children had anemia. Of note, most parts of Tanzania including Nanyumbu and Masasi are endemic to intestinal soil transmitted helminths which are also among the major causes of anemia in children living in tropical countries. Likewise, malnutrition and red blood cell disorders including sickle cell anemia are other factors that may have contributed to the observed high prevalence of anemia in non-malaria infected children [49].

Despite the strength of this study, its limitations include: the children were not screened for SHTs which are also a significant cause of anemia in tropical countries. Red blood cells disorders were also not assessed and are known to cause anemia. Nutritional assessment was performed, however will be presented elsewhere. Mosquito abundance was not assessed, and this could have probably explained the significant differences in malaria prevalence between Masasi and Nanyumbu districts. Nonetheless, the authors believe that these limitations have not affected the validity of the study findings.

# Conclusion

The prevalence of malaria and anemia was high among underfive children in Masasi and Nanyumbu districts and was strongly associated with household socioeconomic factors. Improving the household socioeconomic status is expected to reduce the prevalence of malaria and anemia in the study area.

# Acknowledgement

The authors extend their appreciation to the children and parents/guardians for participating in the study. We would also like to extend our appreciation to the local authorities for their support in all steps of this study.

# References

1.	WHO. World Malaria Report 2015. World Health Organization, Geneva, Switzerland. 2015.

2.	WHO. World Malaria Report 2017. World Health Organization, Geneva, Switzerland. 2017.

3.	WHO. World Malaria Report 2010. World Health Organization. Geneva, Switzerland. 2010.

4.	WHO. World malaria report 2020. World Health Organization. Geneva, Switzerland. 2020.

5.	Ghosh K, Ghosh K. Pathogenesis of anemia in malaria: A concise review. *Parasitol Res*. 2007;101:1463–9.

6.	Weatherall DJ, Miller LH, Baruch DI, Marsh K, Doumbo OK, Casals-Pascual C, et al. Malaria and the red cell. *Hematology*. 2002;415:35–57.

7.	White NJ. Anaemia and malaria. *Malar J.* 2018;17:317.

8.	Murphy SC, Breman JG. Gaps in the childhood malaria burden in Africa: Cerebral malaria, neurological sequelae, anemia, respiratory distress, hypoglycemia, and complications of pregnancy. *Am J Trop Med Hyg*. 2001;64:57–67.

9.	Ehrhardt S, Burchard GO, Mantel C, Cramer JP, Kaiser S, Kubo M, et al. Malaria, anemia, and malnutrition in African children - Defining intervention priorities. *J Infect Dis*. 2006;194:108–14.

10.	Haldar K, Mohandas N. Malaria, erythrocytic infection and anemia. *Am Soc Hematol Educ Program.* 2009; 574: 87–93.

11.	Papaioannou I, Utzinger J, Vounatsou P. Malaria-anemia comorbidity prevalence as a measure of malaria-related deaths in sub-Saharan Africa. *Sci Rep*. 2019;9:11323.

12.	Edwards HM, Sriwichai P, Kirabittir K, Prachumsri J, Chavez IF, Hii J. Transmission risk beyond the village: entomological and human factors contributing to residual malaria transmission in an area approaching malaria elimination on the Thailand – Myanmar border. *Malar J*. 2019;18:221.

13.	Khatib RA, Chaki PP, Wang DQ, Mlacha YP, Mihayo MG, Gavana T, et al. Epidemiological characterization of malaria in rural southern Tanzania following China-Tanzania pilot joint malaria control baseline survey. *Malar J*. 2018;17:292.

14.	Vajda ÉA, Webb CE. Assessing the Risk Factors Associated with Malaria in the Highlands of Ethiopia: What Do We Need to Know ? *Trop Med Infect Dis*. 2017;2:4.

15.	Patz JA, Olson SH. Malaria risk and temperature: Influences from global climate change and local land use practices. *PNAS*. 2006;103(15):5635–6.

16.	Sultana M, Sheikh N, Mahumud RA, Jahir T, Islam Z. Prevalence and associated determinants of malaria parasites among Kenyan children. *Trop Med Heal*. 2017;45:25.

17.	West PA, Protopopoff N, Rowland M, Cumming E, Rand A, Drakeley C, et al. Malaria Risk Factors in North West Tanzania: The Effect of Spraying, Nets and Wealth. *PLoS One*. 2013;8(6):e65787.

18.	Chirebvu E, Chimbari MJ, Ngwenya BN. Assessment of Risk Factors Associated with Malaria Transmission in Tubu Village, Northern Botswana. Hindawi. 2014;2014. e403069.

19.	Oladeinde BH, Omoregie R, Olley M, Anunibe JA, Onifade AA. Malaria and Anemia among Children in a Low Resource Setting In Nigeria. *Iran J Parasitol*. 2012;7:3:31–7.

20.	NBS. Malaria Indicator Survey 2017. National Beaural of Statistics, Dodoma, Tanzania. 2017.

21.	NMCP. National malaria stratergic plan 2014-2020. National Malaria Control Prograamme. Ministry of Health and Social Welfare. Dar es salaam, The United Republic of Tanzania. 2014.

22.	PMI. Tanzania malaria operational plan fiscal year 2014. President’s malaria initiative. Dar es Salaam, Tanzania. 2014.

23.	PMI. Tanzania malaria operational plan fiscal year 2019. President’s Malaria Initiative. Dar es Salaam, Tanzania. 2019.

24.	PMI. Tanzania malaria operational plan fiscal year 2017. President’s Malaria Initiative. Dar es Salaam, Tanzania. 2017.

25.	WHO. Universal access to core malaria interventions in high-burden countries. World Health Organisation. Geneva, Switzerland. 2018.

26.	Guerra M, Sousa B De, Mabale NN, Berzosa P, Arez AP. Malaria determining risk factors at the household level in two rural villages of mainland Equatorial Guinea. *Malar J*. 2018;17:203.

27.	Mazigo HD, Obasy E, Mauka W, Manyiri P, Zinga M, Kweka EJ, et al. Knowledge, Attitudes, and Practices about Malaria and Its Control in Rural Northwest Tanzania. *Malar Res Treat.* 2010; 2010. e794261.

28.	Somi MF, Butler JRG, Vahid F, Njau J, Kachur SP, Abdulla S. Is there evidence for dual causation between malaria and socioeconomic status? Findings from rural Tanzania. *Am J Trop Med Hyg.* 2007;77:1020–7.

29.	Lowassa A, Mazigo HD, Mahande AM, Mwangonde BJ, Msangi S, Mahande MJ, et al. Social economic factors and malaria transmission in Lower Moshi, Northern Tanzania. *Parasites and Vectors*. 2012;5:129.

30.	Mmbando BP, Segeja MD, Msangeni HA, Sembuche SH, Ishengoma DS, Seth MD, et al. Epidemiology of malaria in an area prepared for clinical trials in Korogwe, north-eastern Tanzania. *Malar J*. 2009;8:165.

31.	Coleman M, Coleman M, Mabaso MLH, Mabuza AM, Kok G, Coetzee M, et al. Household and microeconomic factors associated with malaria in Mpumalanga, South Africa. *Trans R Soc Trop Med Hyg*. 2010;104(2):143–7.

32.	Sakwe N, Bigoga J, Ngondi J, Njeambosay B, Esemu L, Kouambeng C, et al. Relationship between malaria, anaemia, nutritional and socio-economic status amongst under-ten children, in the North Region of Cameroon: A cross-sectional assessment. *PLoS One.* 2019;14(6): e0218442.

33.	Deressa W, Ali A, Berhane Y. Household and socioeconomic factors associated with childhood febrile illnesses and treatment seeking behaviour in an area of epidemic malaria in rural Ethiopia. *Trans R Soc Trop Med Hyg*. 2007;101(9):939–47.

34.	Ayele DG, Zewotir TT, Mwambi HG. Prevalence and risk factors of malaria in Ethiopia. *Malar J*. 2012;11:195.

35.	Custodio E, Descalzo MÁ, Villamor E, Molina L, Snchez I, Lwanga M, et al. Nutritional and socio-economic factors associated with *Plasmodium falciparum* infection in children from Equatorial Guinea: Results from a nationally representative survey. *Malar J*. 2009;8:225.

36.	Yadav K, Dhiman S, Rabha B, Saikia PK, Veer V. Socio-economic determinants for malaria transmission risk in an endemic primary health centre in Assam, India. *Infect Dis Poverty*. 2014;3:19.

37.	Ong’echa JM, Keller CC, Were T, Ouma C, Otieno RO, Landis-Lewis Z, et al. Parasitemia, anemia, and malarial anemia in infants and young children in a rural holoendemic Plasmodium falciparum transmission area. *Am J Trop Med Hyg*. 2006;74(3):376–85.

38.	Gahutu JB, Steininger C, Shyirambere C, Zeile I, Cwinya-Ay N, Danquah I, et al. Prevalence and risk factors of malaria among children in southern highland Rwanda. *Malar J*. 2011;10:134.

39.	Kateera F, Ingabire CM, Hakizimana E, Kalinda P, Mens PF, Grobusch MP, et al. Malaria, anaemia and under-nutrition: Three frequently co-existing conditions among preschool children in rural Rwanda. *Malar J*. 2015;14:440.

40.	Gillies MT. The role of carbon dioxide in host-finding by mosquitoes (Diptera : Culicidae): a review. *Bull eni Res*. 1980;70:525–32.

41.	Spitzen J, Smallegange RC, Takken W. Effect of human odours and positioning of CO2 release point on trap catches of the malaria mosquito *Anopheles gambiae sensu stricto* in an olfactometer. *Physiol Entomol*. 2008;33(2):116–22.

42.	Ghebreyesus TA, Haile M, Witten KH, Getachew A, Yohannes M, Lindsay SW, et al. Household risk factors for malaria among children in the Ethiopian highlands. *Trans R Soc Trop Med Hyg*. 2000;94(1):17–21.

43.	Ronald LA, Kenny SL, Klinkenberg E, Akoto AO, Boakye I, Barnish G, et al. Malaria and anaemia among children in two communities of Kumasi, Ghana: A cross-sectional survey. *Malar J*. 2006;5:105.

44.	Klinkenberg E, McCall PJ, Wilson MD, Akoto AO, Amerasinghe FP, Bates I, et al. Urban malaria and anaemia in children: A cross-sectional survey in two cities of Ghana. *Trop Med Int Heal*. 2006;11(5):578–88.

45.	Dobbs KR, Dent AE. *Plasmodium* malaria and antimalarial antibodies in the first year of life. *Parasitology*. 2016;143(2):129–38.

46.	Reynaldi A, Dent AE, Schlub TE, Ogolla S, Rochford R, Davenport MP. Interaction between maternally derived antibodies and heterogeneity in exposure combined to determine time-to-first *Plasmodium falciparum* infection in Kenyan infants. *Malar J*. 2019;18:19.

47.	Mazigo HD, Meza W, Ambrose EE, Kidenya BR, Kweka EJ. Confirmed malaria cases among children under five with fever and history of fever in rural western Tanzania. BMC Res Notes. 2011;4:359.

48.	Tahita MC, Tinto H, Menten J, Ouedraogo JB, Guiguemde RT, Van Geertruyden JP, et al. Clinical signs and symptoms cannot reliably predict *Plasmodium falciparum* malaria infection in pregnant women living in an area of high seasonal transmission. *Malar J*. 2013;12:464.

49.	Yeka A, Nankabirwa J, Mpimbaza A, Kigozi R, Arinaitwe E, Drakeley C, et al. Factors associated with malaria parasitemia, anemia and serological responses in a spectrum of epidemiological settings in Uganda. *PLoS One*. 2015;10(3): e0118901.

Authors’ contribution
AM, BPM, BN, FC, FM, ROM, SL, and SM provided the conception and design of the study. BPM, ROM and BS collected data in the field. BPM, BN and ROM performed data analysis and interpretation. ROM drafted the manuscript together with BPM and BN. All authors revised the manuscript critically for intellectual content and approved the final version.

# Availability of data and material

All relevant data are within the manuscript and its supporting information files.

# Authors’ information

Department of Microbiology, Immunology and Parasitology, Hubert Kairuki Memorial University, Dar es Salaam, Tanzania.

Richard O. Mwaiswelo

Departments of Statistics, National Institute for Medical Research, Tanga Centre, Tanga, Tanzania.
Bruno P. Mmbando

National Malaria Control Programme, Ministry of Health, Community Development, Gender, Elderly and Children, Dodoma, Tanzania.

Frank Chacky, Fabrizio Molteni, Ally Mohamed, and Samwel Lazaro

Department of Medical Parasitology and Entomology, Muhimbili University of Health and Allied Sciences, Dar es Salaam, Tanzania

Billy Ngasala and Bushukatale Samuel

Directorate of Research, Coordination and Promotion, Tanzania Commission of Science and Technology, Dar es Salaam, Tanzania.

Sylvia F. Mkalla

# Abbreviations

ACT: Artemisinin based combination therapy

CHWs: Community Health Workers

CI: Confidence Interval

GPS: Global Positioning System

Hb: Haemoglobin

IQR: Interquartile range
ITN: Insecticide Treated Nets

mRDT: Malaria Rapid Diagnostic Test
NIMR: National Institute for Medical Research
ODK: Open Data Kit
QGIS: Quantum Geographical Information System
SD: Standard deviation

SMC: Seasonal Malaria Chemoprevention

SSA: Sub-Saharan Africa

WBC: White Blood Cells

# Funding

The study was funded by Global Fund.
