# Supplementary material for: Malaria infection and anemia status in under-five children from Southern Tanzania where seasonal malaria chemoprevention is being implemented
Source: PLoS One. 2021 Dec 2;16(12):e0260785. doi: 10.1371/journal.pone.0260785 (PMC8638878; doi:10.1371/journal.pone.0260785)
Supplement: Attachment — Submitted filename: Response to Reviewers.docx [file pone.0260785.s004.docx]

**Response to Reviewers**

**A: Academic Editor**

1. Acknowledgments/ Funding Section of your manuscript: The study was funded by Global Fund.

**Response:** The statement has been removed from the manuscript as instructed, and it has now been added through the online portal.

2. No authors have competing interests. Please complete your Competing Interests on the online submission form to state any Competing Interests.

**Response:** Done

3. PLOS requires an ORCID iD for the corresponding author in Editorial Manager on papers submitted after December 6th, 2016. Please ensure that you have an ORCID iD and that it is validated in Editorial Manager.

**Response:** ORCID iD has been validated

**B: Reviewer #1**

My compliments to the authors, this is a well written manuscript about a properly designed and explained study. However, I have a few questions/remarks though:

1. **Title**: The title was not fluent. So I wanted to suggest “Malaria infection and anemia status in underfive South Tanzanian children where seasonal malaria chemoprevention is being implemented” instead of “Malaria infection and anemia status in underfive children in an area initiating seasonal malaria chemoprevention in Southern Tanzania”.

**Response:** The title suggested by the reviewer has been taken as it is, thank you.

2. Under introduction
   Justification and rationale for the study were not clearly stated. However, the introduction was more of information from literature. Please kindly review your introduction by elaborating on your justifications and rationales.

**Response:** Elaboration on justifications and rationales has been given, Lines 70-71, and 106-113.

3. Was the Ward randomization performed with a computer? Please elaborate.

**Response:** A computer software, Research Randomizer was used to randomize wards. Line 149-150.

4. Under study population

- I will suggest you rephrase it (P6, line 127) to mean willingness of the parents or caregivers to consent since these infants and your children are ethnically ineligible to consent for the study themselves.

**Response:** The sentence has been rephrased by adding the parents or caregivers, to indicate the willingness of parents or caregivers. Line 165.

- P6, line 128, please add the duration of stay in the study area.

**Response:** The duration has been added, the family should have lived in the study area for least 5 years. Line 168-69.

- Under malariometric study
  - Please cite (P7, lines 148-149).

**Response:** The references number 29 and 30 have been added.

- Please specify the number of readings used in the calculation for average parasite density (P8, lines 170- 171).

**Response:** The number of the readings used in the calculation for average parasite density has been explained, and now it reads, “the average parasite density of three readings was used in case of difference in parasite density of greater than 30%, whereas the average of the two positive readings was used for the case of positive versus negative results”. Lines 215-16.

- Please cite (P8, lines 174-175).

**Response:** Reference number 31 has been added.

- Under ethical consideration
  - Please cite (P8 line 194-195).

**Response:** Reference number 32 has been added.

- Under relationship between clinical presentation and malaria infection status
  - The sentence is not clear (P12, lines 259-263).

**Response:** The sentence has been rephrased as suggested. Please clearly state whether it is positively or negatively associated (P13, line 266).

**Response:** Positively associated, and the word has been added in the sentence. Line 315.

- See Attachment for others minor errors.

**Response:** All the minor errors have been corrected.

**C: Reviewer #2:**

Congratulations on the manuscript and the intense work carried out in the field.

**Response:** Thank you for the compliment.
