# Supplementary material for: Malaria infection and anemia status in under-five children from Southern Tanzania where seasonal malaria chemoprevention is being implemented
Source: PLoS One. 2021 Dec 2;16(12):e0260785. doi: 10.1371/journal.pone.0260785 (PMC8638878; doi:10.1371/journal.pone.0260785)
Supplement: Attachment — Submitted filename: Response to Reviewers .docx [file pone.0260785.s005.docx]

**Response to Reviewers**

**Editor**

Please review your reference list to ensure that it is complete and correct.

**Response:**

The reference list has been reviewed, it is complete and correct.

**Reviewer #2:**

1. Dear author, I guess you might not have seen the final parts of my comments, so it was not approached by you in the answer. I am copying it right here again. It is very simple one.

It needs a better discussion about the presence of interfering or confounding variables in the results or even the complex causal relationship between socioeconomic data and malaria and anemia. When, socioeconomic conditions can be the cause of malaria and anemia, but also malaria and anemia are causes of low socioeconomic condition.

**Response:**

The discussion on causal relationship between socioeconomic data and malaria and anemia have been added on lines 330-333, 339-343, 385-389, 393-399.

2. The manuscript needs language corrections, mainly in the introduction and methodology.(I believe, this one has already been settle).

**Response**

Done.
